# Entanglement and Fisher Information for Atoms–Field System in the Presence of Negative Binomial States

**DOI:** 10.3390/e24121817

**Published:** 2022-12-13

**Authors:** Kamal Berrada, Sayed Abdel-Khalek, Mariam Algarni, Hichem Eleuch

**Affiliations:** 1 Department of Physics, College of Science, Imam Mohammad Ibn Saud Islamic University (IMSIU), P.O. Box 5701, Riyadh 11432, Saudi Arabia; 2 Department of Mathematics and Statistics, College of Science, Taif University, P.O. Box 11099, Taif 21944, Saudi Arabia; 3 Department of Mathematics, Faculty of Science, Sohag University, Sohag 82524, Egypt; 4 Department of Mathematical Sciences, College of Science, Princess Nourah bint Abdulrahman University, P.O. Box 84428, Riyadh 11671, Saudi Arabia; 5 Department of Applied Physics and Astronomy, University of Sharjah, Sharjah P.O. Box 27272, United Arab Emirates; 6 College of Arts and Sciences, Abu Dhabi University, Abu Dhabi 59911, United Arab Emirates; 7 Institute for Quantum Science and Engineering, Texas A&M University, College Station, TX 77843, USA

**Keywords:** negative binomial states, binomial distribution, atomic systems, entanglement, parameter estimation

## Abstract

We developed a quantum scheme of two atoms (TAs) and field initially in a negative binomial state (NBS). We displayed and discussed the physical implications of the obtained results in terms of the physical parameters of the model. By considering that the TAs were initially prepared in a maximally entangled state, and that the single-mode field was in the NBS, the dynamics of quantum phenomena such TAs–field entanglement, TAs entanglement, and parameter estimation were examined. We found that the quantum quantifiers exhibited randomly quasi-periodic and periodic oscillations that depended on the success probability, photon number transition, and the intensity-dependent coupling effect. Furthermore, we analyzed the connection between the dynamical behavior of the quantifiers. This system can be compared with some other ones that are being discussed in the literature, in order to realize the quantum entanglement, and to control the precision of the parameter estimation.

## 1. Introduction

The simplest physical model that represents a two-level system coupled with a quantized field is the Jaynes–Cummings model (JCM), which was first proposed by Jaynes and Cummings [1] in 1963. In the JCM, the field was examined in the context of quantum mechanics, in contrast to the methods in which the field was classically processed. For short time intervals, Cummings noted evidence of coherence oscillation and decay of the oscillation amplitude in this model [2]. On the other hand, it was shown for larger times that there were succeeding revivals and periodic collapses of the atomic population [3]. The collapse and revival phenomena in the JCM have been verified experimentally [4]. The entanglement nature of the atom–photon state in the JCM was investigated in [5]. The dynamics of the atomic density operator of a dissipative JCM was examined in [6]. In that paper, the authors also provided the limit for obtaining the state with maximum value of von Neumann entropy. The dynamics of quantum entanglement for a double JCM was examined in [7,8,9,10,11,12,13]. The entanglement phenomenon of sudden death in the double JCM was observed [14]. For further studies, see [15,16,17,18,19,20,21,22]. Since the JCM is considered an ideal in the context of quantum optics, its extension has been considered, such as in the case of a single-mode field with multi-level atoms and the Tavis–Cummings model [23,24,25,26,27,28].

Quantum entanglement is a type of nonlocal correlation which is considered an important concept in the theory of quantum mechanics; it exhibits characteristics that differentiate quantum systems from their classical counterparts [29,30]. In recent decades, the phenomenon of quantum entanglement has become a powerful source at the core of quantum technologies, and has contributed to the development of many areas of physics, such as quantum thermodynamics [31,32], quantum metrology [33,34,35], and physics of the solid state [36,37,38,39,40]. The characterization and quantification of quantum entanglement has aroused great research interest [29,30,41]. Recent advances in the technology of quantum information have provided more information and increased awareness of nonlocal correlation. Considerable physical phenomena, such as sudden birth of entanglement and sudden death of entanglement, have been investigated [42,43]. Therefore, the processing and transmission of information during quantum dynamics is limited by the decoherence effect, where the discussion of dynamical decay and studies of entanglement stability become very important.

Several generalizations of quantum Fisher information have been proposed so far, such as for quantum many-body systems [44,45] and multipartite quantum Fisher information (QFI) [46,47,48]. The connection between the quantum Fisher information dynamical properties and the tomographic entropy of a single atom and field in an excited binomial field state has been studied [49]. It is also worth noting that the QFI related to quantum metrology is tied to local quantum uncertainty. According to the dynamics of a quantum state, it has been shown that the skew information is the bound of the QFI in terms of the phase shift parameter [50]. By exploring the concept of the quantum uncertainty on an observable, a class of measures of quantum correlations of bipartite systems was investigated [51]. Using QFI, Kim and et al. developed the concept of local QFI as a discord-like measure of nonclassical correlations [52]. It was discovered that the local QFI is a crucial tool for learning how nonclassical correlations can enhance the precision and effectiveness of quantum metrology protocols. The dynamics of the local quantum uncertainty has been examined [53]. The purpose of this research was to develop a quantum scheme based on the Tavis–Cummings model, of TAs and field initially in an NBS. By considering that the TAs were initially prepared in a maximally entangled state and the single-mode field was in the NBS, we analyzed the dynamics of TAs–field entanglement, TAs entanglement, and parameter estimation.

Radiation fields with nonclassical states, such as coherent states, number states, and phase states, have been extensively discussed; they have played an essential role since the early days of quantum optics, with many approaches for generating these states [54]. The binomial states were introduced as states that interpolate between the most classical coherent states and the most nonclassical number states [55,56,57,58]. They share the characteristics of both and reduce each to different limits, and they are defined as a linear combination of the number states of the harmonic oscillator, with coefficients chosen such that the photon probability distribution is binomial. The binomial states cannot exhibit the minimum uncertainty product for any such finite combination [59]. Some properties of these field states, including their interaction with atom systems as well as methods of their generation, were discussed in the literature [60,61,62,63]. Furthermore, it has been shown that the binomial states may display distinct nonclassical properties, exhibiting sub-Poissonian and antibunching behaviors [55]. The generalization of the concept of the binomial states to the squeezed states [64], hypergeometric state [65], deformed states [66], and number-phase states [67] has been explored. On the other side, the photon number distribution with negative binomial distribution is considered in the context of negative binomial states (NBSs) [68,69]. These states are different from the binomial states. The NBSs are considered to be the intermediate phase-coherent states tending to coherent states and Susskind–Glogower states in two different limits. It has been proven that the NBSs can represent su(1,1) coherent states via Holstein Primakoff realization, and exhibit strong squeezing effects [68].

The remainder of the manuscript is structured as follows. In Section 2, we describe the physical model and its solution. Section 3 introduces the measures of quantumness and its essential concepts. In Section 4, we present and discuss the numerical results. Finally, we summarize the research in Section 5.

## 2. Hamiltonian and Dynamics

The proposed Hamiltonian of the system describing the interaction of the TAs, where each atom has the upper and lower state that are identified by $|0_{j}\rangle \;\mathrm{and}\;\langle 1_{j}|$, respectively, and single-mode field is given by the following:

(1) $${\hat{H}}_{TLAs-\mathrm{Field}}^{j}=\eta \left\{{\hat{a}}^{k}f\left({\hat{a}}^{†}\hat{a}\right)|0_{j}\rangle \langle 1_{j}|+f\left({\hat{a}}^{†}\hat{a}\right){\hat{a}}^{{†}^{k}}|1_{j}\rangle \langle 0_{j}|\right\},$$

where k is the number of contributing photons between the field and Tas, with η describing the TAs–field coupling, and f is the intensity-dependent coupling. The operators ${\;\hat{a}\;\mathrm{and}\;\hat{a}}^{†}$, respectively, indicate the annihilation and creation operators of the single mode field.

The wave function corresponding to the interaction Hamiltonian is as follows:

(2) $$\left|\Upsilon \left(T\right)\right.\rangle =\exp\left[-iT\left\{{\hat{H}}_{TAs-\mathrm{Field}}^{1}+{\hat{H}}_{TAs-\mathrm{Field}}^{2}\right\}\right]\left|\Upsilon \left(0\right)\right.\rangle ,$$

where we have assumed that T=ηt is the scaled time. The system wave function at T=0 is considered to be the following:

(3) $$|\Upsilon \left(0\right)\rangle =|\Upsilon_{\mathrm{TIAs}}\left(0\right)\rangle ⊗|\Upsilon_{\mathrm{Field}}\left(0\right)\rangle =\frac{1}{\sqrt{2}}\left(|0_{1}0_{2}\rangle +|1_{1}1_{2}\rangle \right)⊗|p;M\rangle ,\;$$

where the initial state of the field is defined in an NBS as [68,69] follows:

(4) $$|p;M\rangle =\sum_{n=0}^{\infty }\sqrt{B_{n}\left(p,M\right)}|n\rangle ,$$

with

(5) $$B_{n}\left(p;M\right)=\left(\begin{array}{c} M+n-1\\ n \end{array}\right)p^{n}{\left(1-p\right)}^{M},\;n=0,1,\cdot \cdot \cdot \;.\;$$

where parameter M is a fixed nonnegative integer, and p is the probability of success satisfying 0<p<1. The photon distribution in the NBS has the form $\langle n|p;M\rangle =B_{n}\left(p;M\right)$. 

At any time T>0, the final TAs–field state is formulated in the following form:

(6) $$|\Upsilon \left(T\right)\rangle =\overset{4}{\underset{j=1}{\sum }}\left(\Lambda_{1}\left(n,T\right)|n,0_{1}0_{2}\rangle +\Lambda_{2}\left(n,T\right)|n+k,0_{1}1_{2}\rangle +\Lambda_{3}\left(n,T\right)|n+k,0_{2}0_{1}\rangle +\Lambda_{4}\left(n,T\right)|n+2k,1_{1}1_{2}\rangle \right)$$

In reliance to the evolution of the density matrix $\varrho_{\mathrm{TAs}-\mathrm{Field}}\left(T\right)=|\Upsilon \left(T\right)\rangle \langle \Upsilon \left(T\right)|$, the reduced matrix of the field and (TAs) denoted by $\varrho_{\mathrm{Field}}\left(T\right)\left(\varrho_{\mathrm{TAs}}\left(T\right)\right)$ is given by the following:

(7) $$\varrho_{\mathrm{TAs}}\left(T\right)={\mathrm{Tr}}_{F}\left\{\varrho_{\mathrm{TAs}-\mathrm{Field}}\left(T\right)\right\}=\overset{4}{\underset{j=1}{\sum }}\overset{4}{\underset{m=1}{\sum }}\varrho_{jm|j\rangle \langle m|},\;$$

(8) $$\varrho_{\mathrm{Field}}\left(T\right)={\mathrm{Tr}}_{\mathrm{TAs}}\left\{\varrho_{\mathrm{TAs}-\mathrm{Field}}\left(T\right)\right\}=\underset{l}{\sum }\varrho_{l}|l\rangle \langle l|.$$

The density matrix elements (7) can be used to evaluate the measures related to the TAs entanglement, TAs-field entanglement, and parameter estimation. 

## 3. Quantumness Measures 

Here, we discuss the temporal behavior of the proposed quantifiers under consideration which provide the atom–field entanglement, TAs entanglement, and parameter estimation. In this regard, we introduce the von Neumann entropy to determine atom–field entanglement and the concurrence for Tas entanglement. We consider the QFI to describe the atomic parameter estimation. 

The von Neumann entropy (VNE) Is presented with the TAs density matrix (8), and is given as follows:

(9) $$\;\;\;\;S_{\mathrm{TAs}}\left(T\right)=-\mathrm{Tr}\left\{\varrho_{\mathrm{TAs}}\left(T\right)\ln\left[\varrho_{\mathrm{TAs}}\left(T\right)\right]\right\},$$

Hence, the VNE formula is evaluated in terms of the eigenvalues $\beta_{1,2,3,4}$ of the TAs density matrix as follows:

(10) $$S_{\mathrm{TAs}}\left(T\right)=-\overset{4}{\underset{j=1}{\sum }}\beta_{j}\ln\beta_{j}$$

The concurrence is employed to evaluate the nonlocal correlation between the two qubits. It is defined by [70] as follows:

(11) $$C_{AA}≔\max\left\{0,\mu_{1}-\mu_{2}-\mu_{3}-\mu_{4}\right\}$$

where $\mu_{j}$ defines the eigenvalues given in the decreasing order of $\;\varrho_{AA}{\tilde{\varrho }}_{AA}$, and ${\tilde{\varrho }}_{AA}$ is the density matrix related to $\sigma_{Y}$ (Pauli matrix) and $\varrho_{AA}^{*}$ (conjugate of $\varrho_{\mathrm{QQ}}$) by the following:

(12) $${\tilde{\varrho }}_{AA}≔\left(\sigma_{Y}⊗\sigma_{Y}\right)\varrho_{AA}^{*}\left(\sigma_{Y}⊗\sigma_{Y}\right)$$

The TAs are in a separable state as${\;C}_{AA}=0$, while they are in the maximally entangled state for${\;C}_{AA}=1$. 

The QFI relies on the estimator parameter φ, which is the two-qubit parameter identified by $U_{\;\varphi \;}=\frac{1}{\sqrt{2}}\left[\exp\left(i\varphi \right)|0_{1}0_{2}\rangle \langle 0_{1}0_{2}|+|1_{1}1_{2}\rangle \langle 1_{1}1_{2}|\right]$. Thus, the optimal target state is $U_{\;\varphi \;}\left|\Upsilon \left(0\right)\right.\rangle$ given by the following:

(13) $${\left|\Upsilon \left(0\right)\right.\rangle }_{\mathrm{opt}}=\frac{1}{\sqrt{2}}\left[\exp\left(i\varphi \right)\left|0_{1}0_{2}\right.\rangle \langle 0_{1}0_{2}|+\left|1_{1}1_{2}\right.\rangle \langle 1_{1}1_{2}|\right]⊗|p;M\rangle .\;$$

The QFI is formulated as in [71,72,73]:

(14) $$F_{\mathrm{TAs}}\left(T\right)=\mathrm{tr}\left\{\;\varrho_{\mathrm{TAs}}\left(\varphi ,T\right)R_{\mathrm{TAs}}{\left(\varphi ,T\right)}^{2}\right\},\;$$

where, within the two-atom density operator, $\;\varrho_{\mathrm{TAs}}$ is related with the symmetric logarithmic derivative operator $R\left(\varphi ,T\right)\;$by [34] as follows:

(15) $$2\frac{\partial R_{\mathrm{TAs}}\left(\varphi ,T\right)}{\partial T}=\varrho_{\mathrm{TAs}}\left(\varphi ,T\right)R_{\mathrm{TAs}}\left(\varphi ,T\right)+R_{\mathrm{TAs}}\left(\varphi ,T\right)\;\varrho_{\mathrm{TAs}}\left(\varphi ,T\right).\;$$

For the single-atom system, we use $\;\varrho_{A}=tr_{B}\left\{\;\varrho_{\mathrm{TAs}}\right\}$ and obtain the following:

(16) $$F_{\mathrm{SA}}\left(T\right)=\mathrm{tr}\left\{\;\varrho_{A}\left(\varphi ,T\right)R_{\mathrm{SA}}{\left(\varphi ,T\right)}^{2}\right\}\;$$

(17) $$2\frac{\partial R_{\mathrm{SA}}\left(\beta ,T\right)}{\partial T}=\varrho_{A}\left(\varphi ,T\right)R_{\mathrm{SA}}\left(\varphi ,T\right)+R_{\mathrm{SA}}\left(\varphi ,T\right)\;\varrho_{A}\left(\varphi ,T\right).\;$$

## 4. Results and Discussion

**Figure 1:**
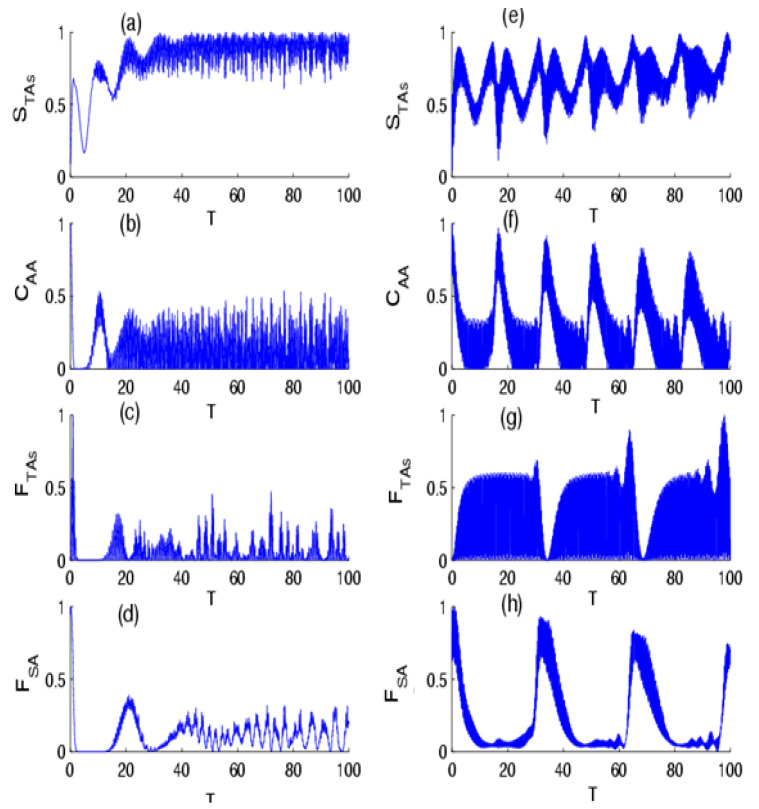
The time evolution of the (**a**) VNE $S_{TAs}$, (**b**) concurrence $C_{AA},$ (**c**) QFI $F_{\mathrm{TAs}},$ and (**d**) QFI $F_{\mathrm{SA}}$ for the scheme of TAs interaction with the field of radiation initially in the NBS with parameter p=1/4 for $f\left(\;\hat{n}\right)=1,\;\mathrm{with}\;$ one photon transition k=1 and M=30. Sub-figures (**e**–**h**) are the same as (**a**–**d**), but for the probability of success parameter p=3/4.

**Figure 2:**
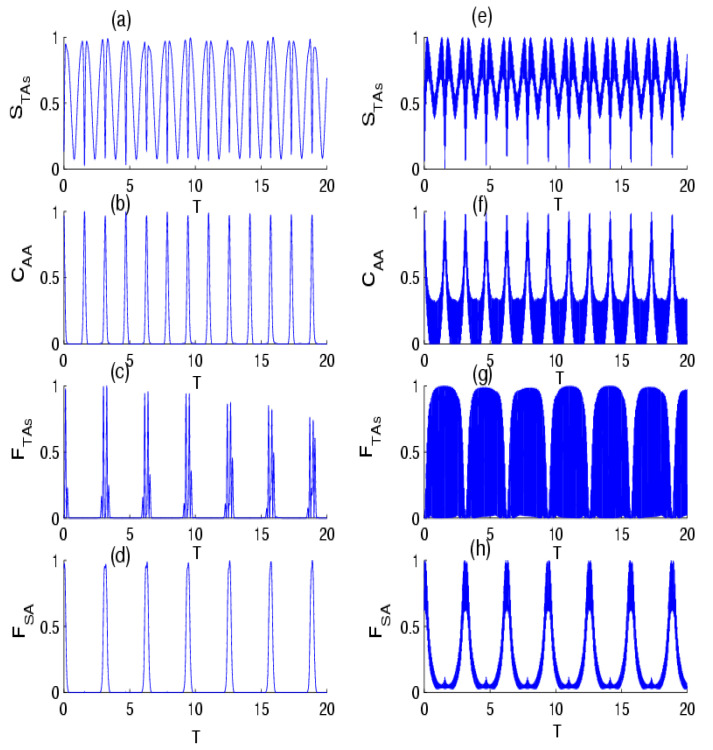
The time evolution of the (**a**) VNE $S_{TAs}$, (**b**) concurrence $C_{AA},$ (**c**) QFI $F_{\mathrm{TAs}},$ and (**d**) QFI $F_{\mathrm{SA}}$ for the scheme of TAs interaction with the field of radiation initially in the NBS with parameter p=1/4 for $f\left(\;\hat{n}\right)=\sqrt{\;\hat{n}},\;\mathrm{with}\;k=1\;\mathrm{and}\;M=30$. Sub-figures (**e**–**h**) are the same as (**a**–**d**), but for the probability of success parameter p=3/4.

**Figure 3:**
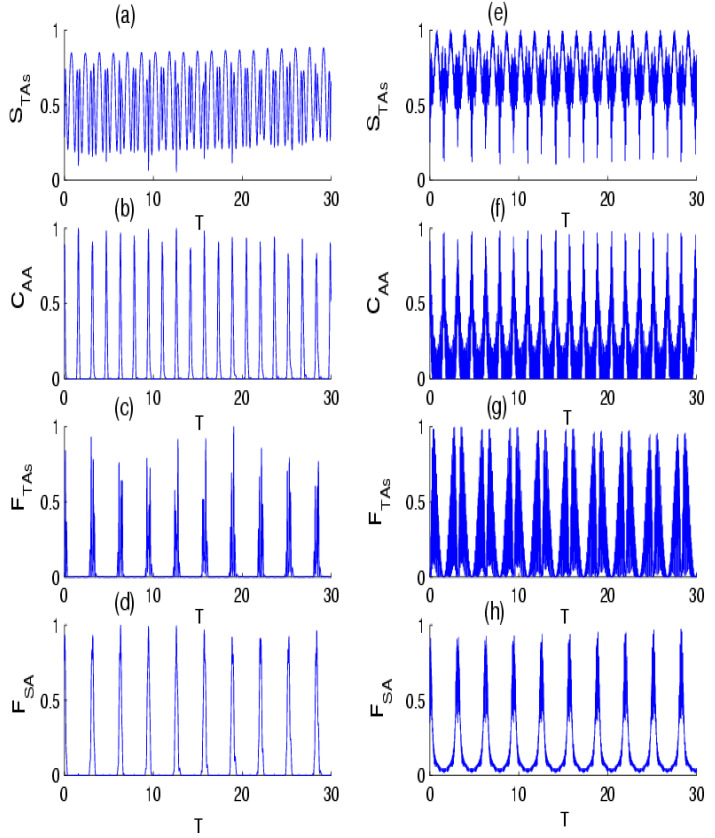
The time evolution of the (**a**) VNE $S_{TAs},$ (**b**) concurrence $C_{AA},$ (**d**) QFI $F_{\mathrm{Tas}}$, and (**d**) QFI $F_{\mathrm{SA}}$ for the scheme of TIAs interaction with the field of radiation initially in the NBS, with parameters p=1/4 for $f\left(\;\hat{n}\right)=1,\;\mathrm{with}\;$ two photon transitions k=2 and M=30. Sub-figures (**e**–**h**) are the same as (**a**–**d**), but for the probability of success parameter p=3/4.

**Figure 4:**
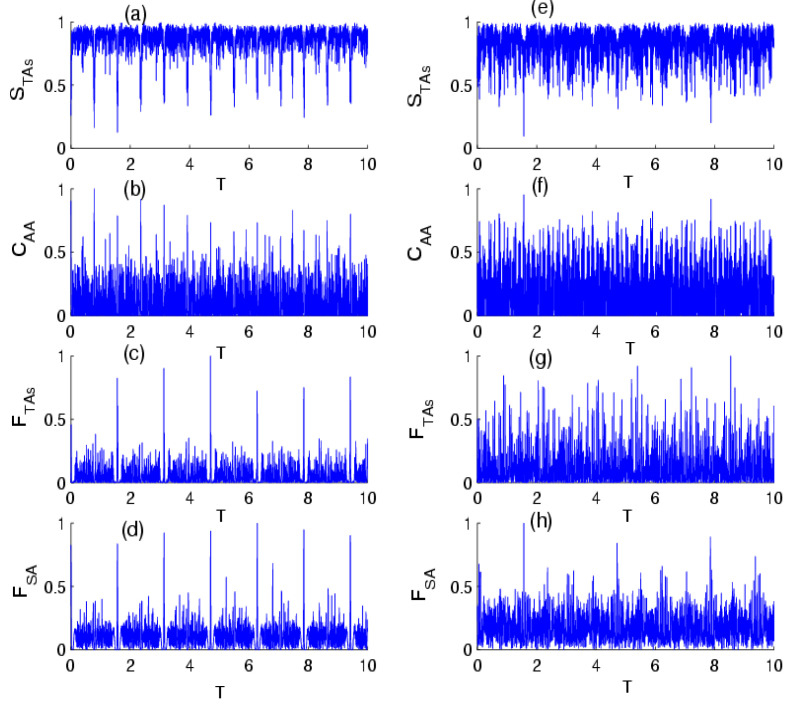
The time evolution of the (**a**) VNE $S_{TAs},$ (**b**) concurrence $C_{AA},$ (**d**) QFI $F_{\mathrm{Tas}}$, and (**d**) QFI $F_{\mathrm{SA}}$ for the scheme of TIAs interaction with the field of radiation initially in the NBS, with parameters p=1/4 for $f\left(\;\hat{n}\right)=\sqrt{\;\hat{n}}\;,\;\mathrm{with}\;$ two photon transitions k=2 and M=30. Sub-figures (**e**–**h**) are the same as (**a**–**d**), but for the probability of success parameter p=3/4.

The numerical results of the quantumness measures in terms of the time T with respect to various physical parameters of the model are shown in Figure 1, Figure 2, Figure 3 and Figure 4, for TAs initially considered in a Bell state, and the field in the context of NBSs. We compared the effects of parameters p and k in both cases, without and with the influence of intensity-dependent coupling. 

In Figure 1, we display the dynamical behavior of the quantifiers in the absence of the effect of intensity-dependent coupling for p=1/4 and p=3/4 with one-photon transition. In general, we noted that the quantifiers exhibited rapid oscillations during the dynamics. The shape of these oscillations is strongly dependent on the value of p. In the case of p=1/4, the VNE that measures the entanglement of the TAs–field state, after suddenly increasing to a local maximum at the beginning of the TAs–field interaction, tends to attain an asymptotic behavior of oscillations. We also observed that the concurrence, which measures the entanglement of the TAs state, decreased from its maximal value and made rapid oscillations. On the other hand, as was seen, the QFI exhibited similar behavior to the TAs–field entanglement according to the physical parameters, with less oscillations in the QFI of a single atom. This indicates that the precision of the parameter estimation in the TAs and TLA state was very sensitive to the TAs–field interaction that resulted in a change in information among subsystems during the time evolution. For the case of p=3/4, we observed that the different quantifiers made quasi-periodic oscillations, where the TAs–field and TAs states became a maximally entangled state, and that QFI reached their maximum value numerous times. Moreover, it can be seen that an increase in the success probability can help realize and stabilize the amount of the TAs–field entanglement, TAs entanglement, and can enhance the accuracy of the parameter estimation during the evolution. In Figure 2, we illustrate the influence of intensity-dependent coupling, $f\left(\;\hat{n}\right)=\sqrt{\;\hat{n}}$, on the temporal evolution of the quantifiers. It is apparent that the impact of intensity-dependent coupling causes the dynamical behavior of the various quantifiers to stabilize with regular oscillations, where their quantifiers made quasi-periodic oscillations accompanied by suppression of the oscillations for p=1/4; however, for p=3/4, their dynamics presented periodic oscillations. 

Figure 3 refers to the influence of the photon-transition parameter on the time evolution of the quantumness measures in the absence of the effect of intensity-dependent coupling for p=1/4 and p=3/4. Generally, quantum quantifiers are strongly affected by the parameter k. In the case of $f\left(\;\hat{n}\right)=1$, the quantifiers represent quasi-periodic oscillations and periodic oscillations. In this limit, the periodicity and maximum values of the quantifiers strictly depend on the parameter p. This shows that a rise in the transition of photons can help to stabilize and realize the degree of the TAs–field entanglement, TAs entanglement, and can enhance the accuracy of the parameter estimation during the evolution. When we compare Figure 3 with Figure 2, the results indicate that the physical parameters acted on the behavior of the quantifiers in similar ways for both cases p=1/4 and p=3/4. In the case of k=2 and $f\left(\;\hat{n}\right)=\sqrt{\;\hat{n}}$, as shown in Figure 4, the quantum quantifiers exhibited quasi-periodic behavior, with rapid oscillation for p=1/4 and a complex structure of oscillations for p=3/4 from the beginning of the TAs–field interaction. From these results, we can deduce that the control of the quantumness measures in the present model can be made by a convenable choice of parameters p and k in the absence and presence of the influence of intensity-dependent coupling, showing the relationship between the measures according to the initial parameters of the model. We note that in the M→∞ and p→0 limits, we recovered a dynamical behavior of the quantifiers that was very similar to the case of the Tavis–Cummings model in the context of Glauber coherent states [74].

## 5. Conclusions

A quantum scheme, based on the Tavis–Cummings model of two atoms (TAs) and field initially in a negative binomial state (NBS), was introduced. The density matrices of the subsystems were obtained explicitly. Physical implications of the obtained results were displayed and discussed in terms of the physical parameters of the model. By considering that the TAs were initially prepared in a Bell state, and the single-mode field was in the NBS, the dynamics of quantum phenomena such TAs–field entanglement, TAs entanglement, and parameter estimation obtained from the whole system density matrix were examined. We demonstrated that the quantum quantifiers exhibited randomly quasi-periodic and periodic oscillations, depending on the success probability, photon number transition, and intensity-dependent coupling effect. We also showed, through the proposed model, that it is possible to realize maximally entangled states and optimal parameter precision with a convenable choice of the physical parameters. Furthermore, we displayed the relationship between the different quantities according to the initial settings of the parameters. We note that in the M→∞ and p→0 limits, we recovered a dynamical behavior of the quantifiers that was very similar to the case of the Tavis–Cummings model in the context of Glauber coherent states. The obtained results indicate that the developed model may be utilized to reduce the noise impact on the quantifiers, suggesting a future examination of the field–atom interaction in the presence of environments with finite temperature, which is essential in the study of quantum optics.

## Data Availability

Not applicable.
